# Relationship between Mediterranean diet and depression in South Korea: the Korea National Health and Nutrition Examination Survey

**DOI:** 10.3389/fnut.2023.1219743

**Published:** 2023-07-05

**Authors:** Yeong-Geon Hwang, Chongwon Pae, Sang-Hyuk Lee, Ki-Hwan Yook, Chun Il Park

**Affiliations:** ^1^Department of Psychiatry, CHA Bundang Medical Center, Seongnam-si, Republic of Korea; ^2^Graduate School of Clinical Counseling Psychology, CHA University, Seongnam-si, Republic of Korea

**Keywords:** depressive symptoms, dietary patterns, KNHANES, Mediterranean diet score, sex difference

## Abstract

**Background:**

Several studies have shown that adherence to the Mediterranean diet is associated with a lower risk of depression; however, little is known about the Asian population. This study investigated the relationship between adherence to the Mediterranean diet and depression in a sample of the South Korean population.

**Methods:**

In total, 5,849 adults from the 2014 and 2016 Korea National Health and Nutrition Examination Surveys were included in the study. The Mediterranean diet adherence was measured using a modified alternate Mediterranean diet score (mMED) developed to adjust for Korean dietary patterns. The mMED scores using the Food Frequency Questionnaire were divided into four categories (0–2, 3–4, 5–6, and 7–9 points). Subjects with depression were defined as having moderate-to-severe depressive symptoms using the Patient Health Questionnaire-9, with a cutoff value of 10. Logistic regression was used to estimate odds ratios (ORs) and 95% confidence intervals (CIs). A subgroup analysis was performed based on sex.

**Results:**

The results of logistic regression analysis indicated that individuals with higher mMED were 42–73% less likely to report depression compared to individuals with the lowest mMED [ORs (95% CIs) =0.58 (0.37–0.90), 0.50 (0.31–0.80), 0.27 (0.15–0.47)] after adjusting for socio-demographic and health-related variables. In women, individuals with mMED of 7–9 had 71% lower odds of depression [ORs (95% CIs): 0.29 (0.13–0.64)]. In men, individuals with mMED of 5–9 had 55% [ORs (95% CIs): 0.45 (0.23–0.91)] to 79% [ORs (95% CIs): 0.21 (0.08–0.57)] lower odds of depression.

**Conclusion:**

This study suggests that adherence to the Mediterranean diet is inversely associated with depression in both men and women among Korean adults. This study provides evidence that a Mediterranean diet is crucial in preventing depressive symptoms in Asian populations.

## 1. Introduction

It is estimated that approximately 256 million adults (5.02%) worldwide suffer from depression in 2019 (1), and the prevalence of depression has increased more than three times during the COVID-19 pandemic (2, 3). Depression is a leading cause of disability and contributes to the overall burden of disease worldwide (4). A longitudinal study demonstrated that depressive symptoms are associated with an approximately 8-fold increased risk of suicide attempts (5). Furthermore, South Korea has the highest suicide rate among Organization for Economic Cooperation and Development (OECD) countries (6). Effective treatment modalities, including pharmacotherapy for depression, have been established; however, half of the patients with depression show an inadequate response to antidepressants (7). Therefore, developing effective strategies to prevent depression is necessary.

Lifestyle modifications, such as dietary advice or exercise coaching, could be important in preventing depression (8, 9). Particularly, recent systematic reviews and meta-analyses indicated that adhering to a healthy diet can reduce the risk of clinical depression or depressive symptoms (10, 11). Diet quality has been reported to influence several biological processes associated with depression, including the production of monoamine neurotransmitters such as serotonin and dopamine, levels of oxidative stress, brain-derived neurotrophic factor (BDNF), and hypothalamic-pituitary-adrenal (HPA) activity (12).

The Mediterranean diet emerged from a classical study by Keys (13) and has been recognized by the United Nations Educational, Scientific, and Cultural Organization (UNESCO) as an intangible cultural heritage of humanity (14). The Mediterranean diet consists of a daily intake of whole grains, vegetables, legumes, fruits, nuts, dairy products, and olive oil; a weekly intake of fish (often); white meat (moderately); red and processed meat (less often); and moderate amounts of wine (15, 16). Previous evidence has shown that Mediterranean diet adherence is associated with a decreased risk of depression in the Mediterranean and other Western countries, including Greece, Italy, Spain, France, the U.K., the Netherlands, the U.S., and Australia (10, 17–19). Longitudinal studies have shown that the association between diet and depression is not significant in women (20, 21), suggesting sex-based differences.

Among the non-Caucasian population, a cross-sectional study of Iranian adults found an inversely significant association between adherence to a Mediterranean diet and depression (22). The Mediterranean diet index is commonly calculated using the median cutoff from each study sample; therefore, these results may be difficult to apply to other cultures (23). To the best of our knowledge, no evidence exists suggesting that adherence to a Mediterranean diet is associated with depression in Asian populations. Therefore, this study aimed to determine the relationship between adherence to the Mediterranean diet and depression in a nationally representative sample of Korean adults. We also investigated whether the association between depression and Mediterranean diet adherence differed between men and women.

## 2. Materials and methods

### 2.1. Data source and study population

Data were obtained from the National Health and Nutrition Examination Survey of South Korea (KNHANES), conducted by the Korea Centers for Disease Control and Prevention (KCDC). This nationwide survey comprised approximately 10,000 individuals over the age of 1 each year. The objectives of the KNHANES include monitoring trends in health-risk factors and their prevalence. The survey consisted of a health examination, health interview, and nutrition survey conducted by trained medical staff and interviewers. The available database and detailed descriptions can be found on the KNHANES website.^1^

**Figure 1:**
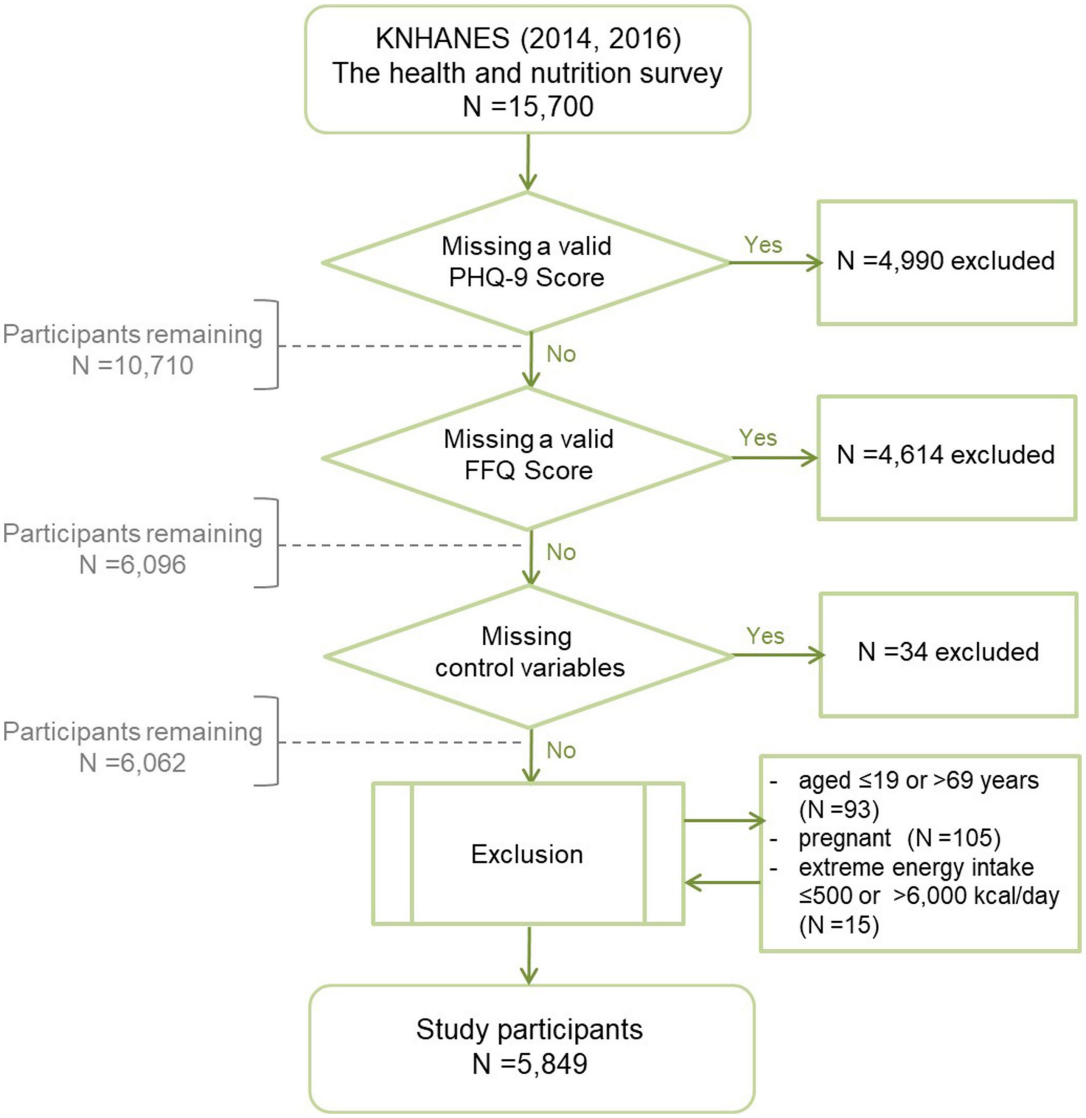
Flowchart of the study participants was presented. KNHANES, Korea National Health and Nutritional Examination Survey; PHQ-9, Patient Health Questionnaire-9; FFQ, Food Frequency Questionnaire.

Our primary sample included 15,700 individuals who completed all three surveys (health examination, health interview, and nutrition survey) in the 2014 and 2016 KNHANES. We excluded individuals with missing valid Patient Health Questionnaire-9 (PHQ-9) scores (*n* = 4,990), Food Frequency Questionnaire (FFQ) scores (*n* = 4,614), or covariate scores (*n* = 34) from this analysis. Individuals who were aged ≤19 or >69 years (*n* = 93), pregnant or breastfeeding (*n* = 105) (24), or had extreme energy intake (≤500 or > 6,000 kcal/day) (*n* = 15) (25) were excluded. The final sample included 5,849 individuals (Figure 1). All participants signed an informed consent form. The KNHANES was reviewed and approved by the KCDC Research Ethics Review Committee, which operates under domestic and international regulations, including the Declaration of Helsinki. The Institutional Review Board (IRB) of CHA Bundang Medical Center of CHA University exempted this IRB review because the databases provided publicly available secondary data (no. 2022-12-051).

### 2.2. Adherence to the Mediterranean diet

Adherence to the Mediterranean diet was measured using the modified alternate Mediterranean diet score (mMED) developed by Kim and Je (25) to adjust for Korean dietary patterns based on the alternate Mediterranean diet score (26). To calculate the mMED, dietary intake was evaluated using semiquantitative FFQ data from the KNHANES from 2012 to 2016. The FFQ consists of 112 items measuring the average frequency of food intake and service size over the past year. Regarding the frequency of food intake, nine categories were general options (from *less than once per month* to *more than three times per day*). For the serving size, the three categories were general options (0.5, 1, or 1.5 being the standard serving size), and the amount of alcoholic beverage was measured using open-ended questions. The consumption of each food item was estimated by multiplying the frequency of intake per week by the serving size (25). The reproducibility of the FFQ was acceptable, ranging from 0.54 to 0.61, and the validity was modest, ranging from 0.29 to 0.45 (27).

The mMED classifies 65 foods into nine groups: vegetables, legumes, fruits, whole grains, red or processed meats, white meat, fish/peanuts, dairy products, and alcohol (25). The mMED was calculated using sex-specific medians for the frequency of intake of each food group as cut-offs. The total mMED ranged from 0 to 9, with each food group scoring either 0 or 1. For vegetables, legumes, fruits, whole grains, white meats, fish/peanuts, and dairy products, the participants were awarded a point if they consumed more than the median intake. For red or processed meat, considering that the intake of red meat is relatively low in Korea (28), a point was awarded if the subjects consumed less than the 75th percentile. Ethanol consumption in alcoholic beverages was calculated according to the 8th Revision Korean Food Composition Table provided by the Korean Nutrition Society (29). A point was awarded for ethanol consumed of 10–50 g and 5–25 g per day for men and women, respectively. Otherwise, zero points were awarded. Finally, mMED was divided into four categories (0–2, 3–4, 5–6, and 7–9 points) for statistical analysis (25).

### 2.3. Depression

The PHQ-9, which has been administered biannually in the KNHANES since 2014, was used to evaluate depression severity (30). The PHQ-9 consists of nine criteria for depressive disorder from the Diagnostic and Statistical Manual of Mental Disorders, 4th Edition (DSM-IV), each rated on a scale from *not at all* (0), *several days* (1), *more than half the days* (2), and *nearly every day* (3), resulting in a total score ranging from 0 to 27. The PHQ-9 cut-point of 10 for major depressive disorder had a high sensitivity (88%) and specificity (88%) (30), and a validation study reported that the Cronbach’s α of the Korean version of PHQ-9 was 0.81 (31). In this study, depression was defined as a PHQ-9 score of 10 (moderate or severe depression) (30).

### 2.4. Covariates

Sociodemographic (sex, age, residential area, household income, marital status, and education) and health-related (sleep, exercise variations, smoking, alcohol use, body mass index, chronic medical diseases, and energy intake) variables were selected as covariates that could affect diet quality or depression.

Household income was classified into quintiles based on the Korean population. Sleep duration was classified as *insufficient (<6 h/day)*, *normal, (<9 h/day)*, and *excessive (≥9 h/day)*. Exercise variation was classified as *none*, *one*, *two, or more*, depending on whether the three types of exercise (strength, aerobic, and walking) were performed at least once a week. Smoking and alcohol use were classified as *current* or *non-use*. Body Mass Index (BMI; kg/m^2^) was classified into four categories: *underweight (BMI < 18.5)*, *normal weight (18.5 ≤ BMI < 23)*, *overweight (23 ≤ BMI < 25)*, and *obesity (25 ≤ BMI)* by calculating the weight and height measured by trained staff. Chronic medical diseases were classified as *none*, *one*, *two, or more*, and diabetes mellitus, hypertension, dyslipidemia, stroke, chronic renal failure, and coronary heart disease, which could directly or indirectly affect depression, were included as comorbidities (32). Energy intake was classified into quartiles according to sex based on the study data.

### 2.5. Statistical analysis

Descriptive statistics were calculated for the frequencies and percentages of sociodemographic and clinical characteristics. Differences between participants’ characteristics were tested using the chi-square test. A False Discovery Rate (FDR) correction was performed to correct for type I error inflation. Using multivariate logistic regression analysis, odds ratios (ORs) and 95% confidence intervals (CIs) for depression were estimated in groups with mMED of 3–4, 5–6, and 7–9 when controlling covariates. Additional analysis was performed to investigate the ORs and 95% CIs for sex differences in the impact on depression using multivariable logistic regression analysis. All data processing and analysis were performed using Python (version 3.9), and a *p*-value < 0.05 was considered significant.

## 3. Results

### 3.1. Description of the study sample

The sociodemographic and clinical characteristics of the 5,849 participants based on the presence of moderate or severe depressive symptoms are summarized in Table 1. Among the participants, the prevalence of depression was 5.5% (4.0% for men and 6.4% for women; more details are presented in Table 1). The results of the chi-square test indicated that the higher the mMED, the lower the frequency of depression [9.7% vs. 6.5% vs. 5.5% vs. 3.3% (FDR-corrected *p* < 0.001)]. The residential area, alcohol consumption, and energy intake were not significantly associated with depression. In contrast, other sociodemographic and health-related variables, including sex, age, household income, marital status, education, sleep, exercise variations, smoking, BMI group, and chronic medical diseases, were significantly associated with depression (FDR-corrected *p* < 0.05).

**Table 1:** Socio-demographics and clinical characteristics of study participants by the presence of depression.

Characteristics	Total (*n* = 5,849)	Depressive (*n* = 319, %)	Non-depressive (*n* = 5,530, %)	*p*-value
**Mediterranean diet score**				**<0.001**
0–2	401	36 (8.98)	365 (91.02)	
3–4	1,684	114 (6.77)	1,570 (93.23)	
5–6	2,174	122 (5.61)	2,052 (94.39)	
7–9	1,590	47 (2.96)	1,543 (97.04)	
**Sex**				**<0.001**
Men	2,265	91 (4.02)	2,174 (95.98)	
Women	3,584	228 (6.36)	3,356 (93.64)	
**Age (years)**				**0.012**
20–29	837	61 (7.29)	776 (92.71)	
30–39	1,404	77 (5.48)	1,327 (94.52)	
40–49	1,467	58 (3.95)	1,409 (96.05)	
50–59	1,458	80 (5.49)	1,378 (94.51)	
60–69	683	43 (6.30)	640 (93.70)	
**Residential area**				0.772
Rural area	947	54 (5.70)	893 (94.30)	
Urban area	4,902	265 (5.41)	4,637 (94.59)	
**Household income**				**<0.001**
Quintile 1	603	93 (15.42)	510 (84.58)	
Quintile 2	1,098	63 (5.74)	1,035 (94.26)	
Quintile 3	1,302	61 (4.69)	1,241 (95.31)	
Quintile 4	1,362	54 (3.96)	1,308 (96.04)	
Quintile 5	1,484	48 (3.23)	1,436 (96.77)	
**Marital status**				**<0.001**
Married	4,301	168 (3.91)	4,133 (96.09)	
Separated/divorced/widowed	1,138	87 (7.64)	1,051 (92.36)	
Never married	410	64 (15.61)	346 (84.39)	
**Education**				**<0.001**
Primary or below	556	61 (10.97)	495 (89.03)	
Middle school	517	34 (6.58)	483 (93.42)	
High school	2,148	112 (5.21)	2,036 (94.79)	
College or above	2,628	112 (4.26)	2,516 (95.74)	
**Sleep**				**<0.001**
Insufficient (<6 h/day)	4,740	215 (4.54)	4,525 (95.46)	
Normal (<9 h/day)	714	78 (10.92)	636 (89.08)	
Excessive (≥9 h/day)	395	26 (6.58)	369 (93.42)	
**Exercise variations**				**<0.001**
None	655	45 (6.67)	610 (93.13)	
One	2,822	180 (6.38)	2,642 (93.62)	
Two or more	2,372	94 (3.96)	2,278 (96.04)	
**Smoking**				**<0.001**
Non-smoker	4,717	216 (4.58)	4,501 (95.42)	
Smoker	1,132	103 (9.10)	1,029 (90.90)	
**Alcohol use**				0.61
No	2,441	138 (5.65)	2,303 (94.35)	
Yes	3,408	181 (5.31)	3,227 (94.69)	
**Body Mass Index group**				**0.021**
Underweight	252	24 (9.52)	228 (90.48)	
Normal weight	2,430	130 (5.35)	2,300 (94.65)	
Overweight	1,303	61 (4.68)	1,242 (95.32)	
Obesity	1864	104 (5.58)	1760 (94.42)	
**Chronic medical disease**				**0.009**
None	4,589	236 (5.14)	4,353 (94.86)	
One	804	44 (5.47)	760 (94.53)	
Two or more	456	39 (8.55)	417 (91.45)	
**Energy intake**				0.051
Quartile 1	1,463	98 (6.70)	1,365 (93.30)	
Quartile 2	1,462	65 (4.45)	1,397 (95.55)	
Quartile 3	1,462	74 (5.06)	1,388 (94.94)	
Quartile 4	1,462	82 (5.61)	1,380 (94.39)	

Depression was defined as a patient health questionnaire-9 score of 10. Bold values were defined as significant at *p*-values < 0.05.

### 3.2. Association between the Mediterranean diet and depression

The results of the multivariate logistic regression analysis of the association between the mMED and depression are presented in Table 2. The mMED significantly decreased the odds of depression [ORs (95% CIs): 0.65 (0.44–0.97), 0.54 (0.36–0.80), 0.32 (0.20–0.50); Model 1]. The association between the mMED and depression remained significant after adjusting socio-demographic variables [ORs (95% CIs): 0.56 (0.37–0.86), 0.50 (0.33–0.77), 0.30 (0.19–0.49); model 2] and all other covariates including sleep, exercise variations, smoking, alcohol use, BMI group, chronic medical disease, and energy intake [ORs (95% CIs): 0.58 (0.37–0.90), 0.50 (0.31–0.80), 0.27 (0.15–0.47); model 3]. This result indicates that individuals with higher mMED were 42 to 73% less likely to report depression compared to those with the lowest mMED.

**Table 2:** Odds ratios and 95% confidence intervals of the multivariable logistic regression for the association between modified Mediterranean diet score (mMED) and depression (PHQ-9 ≥ 10).

	Mediterranean diet score						
	0–2	3–4		5–6		7–9	
	ORs	ORs (95% CIs)	*p*-value	ORs (95% CIs)	*p*-value	ORs (95% CIs)	*p*-value
Model 1^a^	1.0 (ref)	0.65 (0.44–0.97)	0.034	0.54 (0.36–0.80)	0.002	0.32 (0.20–0.50)	<0.001
Model 2^b^	1.0 (ref)	0.56 (0.37–0.86)	0.007	0.50 (0.33–0.77)	0.002	0.30 (0.19–0.49)	<0.001
Model 3^c^	1.0 (ref)	0.58 (0.37–0.90)	0.015	0.50 (0.31–0.80)	0.004	0.27 (0.15–0.47)	<0.001

a Model 1 was unadjusted.

b Model 2 was adjusted for sex, age, residential area, household income, marital status, and educational level.

c Model 3 was additionally adjusted for sleep, exercise variations, smoking, alcohol use, BMI group, chronic medical disease, and energy intake.

Each modified Mediterranean diet score group was compared with the 0–2 group and *p*-value < 0.05 was defined as significant. PHQ-9, patient health questionnaire-9; ORs, odds ratios; CIs, confidence intervals; ref, reference group; BMI, body mass index.

### 3.3. Sex differences in the impact of Mediterranean diet adherence on depression

**Figure 2:**
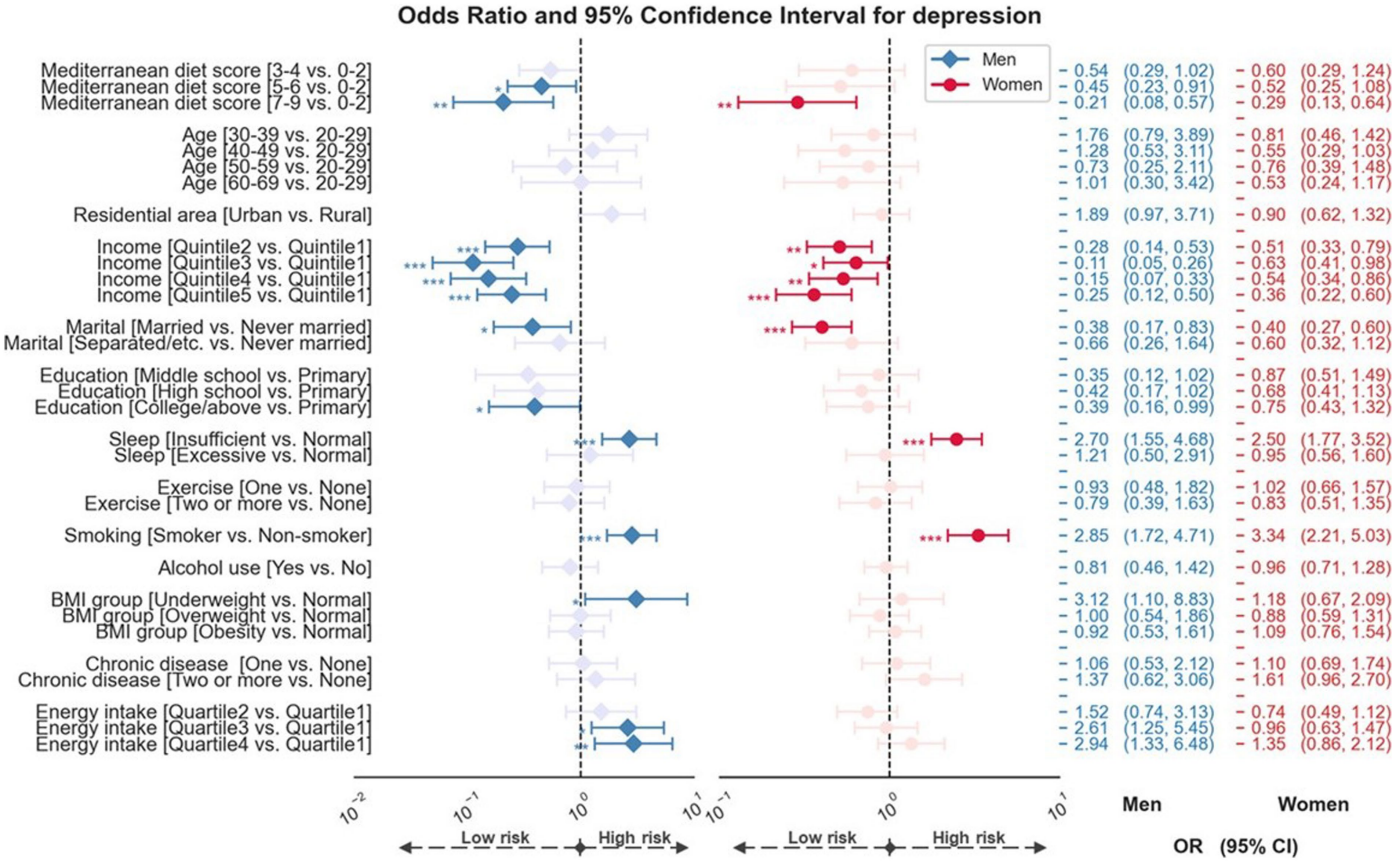
Forest plot showed odds ratios and 95% confidence intervals for sex difference in impact of the Mediterranean diet adherence on depression. Analyses were adjusted for age, residential area, household income, marital status, education, sleep, exercise variations, smoking, alcohol use, BMI group, chronic medical disease, and energy intake. *p*-value < 0.05 was considered to be significant. *, **, and *** denote *p* < 0.05, *p* < 0.01, and *p* < 0.001. OR, odds ratios; CI, confidence intervals; vs., versus; BMI, body mass index.

The results of the multivariate logistic regression analysis for sex differences in the impact of mMED on depression are shown in Figure 2. A significant association was found between depression and mMED after adjusting for all covariates in both men and women. In women, compared to the individuals in the lowest mMED group, individuals with mMED of 7–9 had 71% lower odds of depression [ORs (95% CIs): 0.29 (0.13–0.64)]. In men, individuals with a mMED of 5–9 had a 55% [ORs (95% CIs): 0.45 (0.23–0.91)] to 79% [ORs (95% CIs): 0.21 (0.08–0.57)] lower risk of depression.

Regarding the impact of sociodemographic and health-related factors on depression, men and women who were current smokers or reported having insufficient sleep were more likely to report depression. However, men and women who were married or had higher household incomes were less likely to report depression. Being underweight and high energy consumption were associated with depression only in men.

## 4. Discussion

This study investigated the relationship between adherence to the Mediterranean diet and depression using a representative sample of South Korean adults. We found that individuals with greater adherence to the Mediterranean diet had a lower risk of depression after adjusting for the confounding effects of sociodemographic and health-related variables. Furthermore, a significant association was observed between adherence to Mediterranean diet and depression in both men and women. To the best of our knowledge, this is the first study to examine the relationship between the Mediterranean diet and depression in an Asian population.

Our study found that individuals with greater adherence to the Mediterranean diet had a 42–73% lower risk of depression. This finding was consistent with previous studies suggesting that people with high Mediterranean diet scores were reported to have a 40–45% lower risk of depression (22, 33). Other meta-analysis studies showed adherence to the Mediterranean diet was associated with a 19% lower risk of depression (18, 19). Furthermore, randomized controlled trials have reported that Mediterranean dietary interventions significantly improved depressive symptoms (34, 35). Generally, these consistent results among non-Asian populations suggest the beneficial role of the Mediterranean diet in depression, and our results may confirm the relationship between the Mediterranean diet and depression in Asian cultures.

The mechanism underlying the association between the Mediterranean diet and depression is unclear; however, several potential explanations exist. The Mediterranean diet is rich in vitamin B and omega-3 polyunsaturated fatty acids (PUFA). Vitamin B can be involved in several methylation reactions, including serotonin and other monoamine neurotransmitters, and PUFA can play an important role in central nervous system function and membrane fluidity, which could influence serotonin transport (36). Moreover, fruits, vegetables, and rice wine (37) in the Mediterranean diet may be rich sources of polyphenols with antioxidative and anti-inflammatory effects on depression (38, 39). Polyphenols can also induce BDNF expression by inducing cyclic adenosine monophosphate (cAMP) response element binding (CREB) (40). BDNF activation in the hippocampus and prefrontal cortex plays an important role in regulating depressive symptoms (41). The synergistic effects of these nutrients in the Mediterranean diet may contribute to its protective role against depression. Since this study could not identify the mechanism involved in nutritional biology, further research is needed to identify the mechanisms for the antidepressant properties of the Mediterranean diet.

We conducted further analyses to investigate sex differences in the effects of the Mediterranean diet and other demographic and health-related factors on depression. We found that moderate and high adherence to the Mediterranean diet was associated with a lower risk of depression in men, whereas women showed this relationship only in the high Mediterranean diet group. A previous large prospective cohort study showed that adherence to the Mediterranean diet was associated with incident depression in a male subgroup but not in a female subgroup (20), suggesting that there may be sex differences. Multiple factors, including biological, cultural, and reproductive events, can affect sex differences in the development of depression (42). Particularly, female sex hormones may induce changes in food intake and taste preference (43), as well as affect neuroinflammation and endocrine systems that contribute to depression (44–46). Body image perception by sex can also be considered as another possible explanation (47). Furthermore, longitudinal studies are required to investigate sex differences in the causal relationship between Mediterranean diet adherence and depression in Asian populations. Regarding sociodemographic and health-related factors, in both men and women, the risk of depression was associated with household income, marital status, sleep duration, and smoking, showing a pattern similar to that of previous studies (48–50). Interestingly, the risk of depression was significantly associated only with being underweight in the male group. Considering a previous result that men with depression are more likely to report decreased appetite and weight loss than women (51), low body weight may significantly impact depression in men.

Although this study investigated the adherence to the Mediterranean diet using the mMED index reflecting the Korean food culture, there are some points to consider the characteristics of the Korean dietary situation possibly affecting mental health. For example, most Koreans eat fermented vegetables, including Kimchi and soybean paste. Some studies suggested that fermented food modulates the composition of microbiota, especially via *Lactobacillus* (52, 53). As several studies examined the effect of the microbiome on mental health, such as mood disorders, anxiety disorder, and autism spectrum disorder (54, 55), adherence to fermented food might be linked with mental disorders. Future research investigating the effect of Korean food on depression would elucidate the association between diet and depression in Korean culture.

This study’s key advantage is that it used large and homogeneous national data to analyze the relationship between adherence to the Mediterranean diet and depression among Asians. We used a validated measure of food intake frequency calculated from the Mediterranean diet scoring index based on Korean foods to assess diet adherence (25). Additionally, unlike previous studies on the Mediterranean diet and depression, our findings were adjusted for the effects of various sociodemographic and health-related variables to control for possible confounding effects. However, this study has several limitations. First, this was a cross-sectional study that could not clarify whether adherence to the Mediterranean diet could cause depression. Future prospective studies are required to confirm this causal direction. Second, the FFQ is a self-reported dietary assessment that may be biased toward dietary changes or recall errors. However, the FFQ has been commonly used to measure dietary patterns in epidemiological studies of diet and health and maybe more suitable than other dietary assessment methods for estimating usual intake (56). Third, this study investigated the Mediterranean diet according only to the frequency and amount of food. Therefore, we could not determine the effect of micronutrients. Furthermore, we could not control the impact of behavioral patterns related to food or alcohol consumption including solitary alcohol consumption. Previous studies reported that drinking alone was associated with depression (57, 58). Finally, this study was unable to explain the exact physiological mechanisms underlying the impact of the Mediterranean diet because biomarkers were not used. Future studies should include an assessment of biological mediators to identify the mechanisms by which the Mediterranean diet affects depression.

In summary, our study revealed that adherence to the Mediterranean diet was inversely associated with depression in both women and men in a nationally representative sample of Koreans. This study provides evidence that a Mediterranean diet is crucial in preventing symptoms and supporting positive mental health, even among non-Caucasians.

## Data availability statement

The original contributions presented in the study are included in the article/supplementary material, further inquiries can be directed to the corresponding authors.

## Ethics statement

The studies involving human participants were reviewed and approved by Institutional Review Board of CHA Bundang Medical Center. The ethics committee waived the requirement of written informed consent for participation.

## Author contributions

Y-GH: conceptualization, formal analysis, data curation, and writing of the original draft. CP: methodology and validation. S-HL: supervision, conceptualization, project administration, and funding acquisition. K-HY supervised, validated, wrote, reviewed, and edited the manuscript. CIP: supervision, validation, study design, project administration, funding acquisition, and writing—review and editing. All authors contributed to the article and approved the submitted version.

## Funding

This research was supported by the Basic Science Research Program through the National Research Foundation of Korea (NRF) funded by the Ministry of Education [grant numbers: NRF-2021R1I1A1A01048880 and RS-2023-00238510] and the Ministry of Science and ICT [grant number: NRF-2021M3E5D9025026].

## Conflict of interest

The authors declare that the research was conducted in the absence of any commercial or financial relationships that could be construed as a potential conflict of interest.

## Publisher’s note

All claims expressed in this article are solely those of the authors and do not necessarily represent those of their affiliated organizations, or those of the publisher, the editors and the reviewers. Any product that may be evaluated in this article, or claim that may be made by its manufacturer, is not guaranteed or endorsed by the publisher.

## Footnotes

^1^ https://knhanes.kdca.go.kr

## References

[1] Institute of Health Metrics and Evaluation. Global Health data exchange (Ghdx). (2021). Available at: http://ghdx.healthdata.org/gbd-results-tool?params=gbd-api-2019-permalink/d780dffbe8a381b25e1416884959e88b.

[2] Ebrahimi OV Hoffart A Johnson SU. Physical distancing and mental health during the Covid-19 pandemic: factors associated with psychological symptoms and adherence to pandemic mitigation strategies. Clin Psychol Sci. (2021) 9:489–506. doi: 10.1177/2167702621994545

[3] Ettman CK Abdalla SM Cohen GH Sampson L Vivier PM Galea S. Prevalence of depression symptoms in us adults before and during the Covid-19 pandemic. JAMA Netw Open. (2020) 3:e2019686. doi: 10.1001/jamanetworkopen.2020.19686 32876685 PMC7489837

[4] Collaborators GMD. Global, regional, and National Burden of 12 mental disorders in 204 countries and territories, 1990–2019: a systematic analysis for the global burden of disease study 2019. Lancet Psychiatry. (2022) 9:137–50. doi: 10.1016/S2215-0366(21)00395-3 35026139 PMC8776563

[5] Melhem NM Porta G Oquendo MA Zelazny J Keilp JG Iyengar S. Severity and variability of depression symptoms predicting suicide attempt in high-risk individuals. JAMA Psychiat. (2019) 76:603–13. doi: 10.1001/jamapsychiatry.2018.4513, PMID: 30810713 PMC6551844

[6] OECD. Health at a Glance. OECD indicators. Paris: OECD publishing (2021).

[7] Trivedi MH Rush AJ Wisniewski SR Nierenberg AA Warden D Ritz L. Evaluation of outcomes with citalopram for depression using measurement-based Care in Star* D: implications for clinical practice. Am J Psychiatr. (2006) 163:28–40. doi: 10.1176/appi.ajp.163.1.28, PMID: 16390886

[8] Berk M Sarris J Coulson CE Jacka FN. Lifestyle Management of Unipolar Depression. Acta Psychiatr Scand. (2013) 127:38–54. doi: 10.1111/acps.12124 23586875

[9] Stahl ST Albert SM Dew MA Lockovich MH Reynolds CF III. Coaching in healthy dietary practices in at-risk older adults: a case of indicated depression prevention. Am J Psychiatr. (2014) 171:499–505. doi: 10.1176/appi.ajp.2013.13101373, PMID: 24788282 PMC4083759

[10] Lassale C Batty GD Baghdadli A Jacka F Sánchez-Villegas A Kivimäki M. Healthy dietary indices and risk of depressive outcomes: a systematic review and Meta-analysis of observational studies. Mol Psychiatry. (2019) 24:965–86. doi: 10.1038/s41380-018-0237-8, PMID: 30254236 PMC6755986

[11] Firth J Marx W Dash S Carney R Teasdale SB Solmi M. The effects of dietary improvement on symptoms of depression and anxiety: a Meta-analysis of randomized controlled trials. Psychosom Med. (2019) 81:265–80. doi: 10.1097/PSY.0000000000000673, PMID: 30720698 PMC6455094

[12] Lopresti AL Hood SD Drummond PD. A review of lifestyle factors that contribute to important pathways associated with major depression: diet, sleep and exercise. J Affect Disord. (2013) 148:12–27. doi: 10.1016/j.jad.2013.01.014, PMID: 23415826

[13] Keys A. Coronary heart disease in seven countries. Circulation. (1970) 41:186–95. 5442775

[14] UNESCO. Browse the lists of intangible cultural heritage and the register of good safeguarding practices. (2023). Available at: https://ich.unesco.org/en/lists (Accessed March 3, 2023).

[15] Mediterranean Diet Pyramid. Oldways preservation and exchange trust. (2009). Available at: https://oldwayspt.org/resources/oldways-mediterranean-diet-pyramid (Accessed January 3, 2023).

[16] Bach-Faig A Berry EM Lairon D Reguant J Trichopoulou A Dernini S. Mediterranean diet pyramid today. Science and cultural updates. Public Health Nutr. (2011) 14:2274–84. doi: 10.1017/S1368980011002515, PMID: 22166184

[17] Nicolaou M Colpo M Vermeulen E Elstgeest LE Cabout M Gibson-Smith D. Association of a Priori Dietary Patterns with depressive symptoms: a harmonised Meta-analysis of observational studies. Psychol Med. (2020) 50:1872–83. doi: 10.1017/S0033291719001958, PMID: 31409435 PMC7477372

[18] Shafiei F Salari-Moghaddam A Larijani B Esmaillzadeh A. Adherence to the Mediterranean diet and risk of depression: a systematic review and updated Meta-analysis of observational studies. Nutr Rev. (2019) 77:230–9. doi: 10.1093/nutrit/nuy070, PMID: 30726966

[19] Shafiei F Salari-Moghaddam A Larijani B Esmaillzadeh A. Mediterranean diet and depression: reanalysis of a Meta-analysis. Nutr Rev. (2023): nuad023) 81:889–90. doi: 10.1093/nutrit/nuad023, PMID: 36928725

[20] Adjibade M Assmann KE Andreeva VA Lemogne C Hercberg S Galan P. Prospective association between adherence to the Mediterranean diet and risk of depressive symptoms in the French Su. Vi. Max cohort. Eur J Nutr. (2018) 57:1225–35. doi: 10.1007/s00394-017-1405-3, PMID: 28283824

[21] Lai JS Oldmeadow C Hure AJ McEvoy M Byles J Attia J. Longitudinal diet quality is not associated with depressive symptoms in a cohort of middle-aged Australian women. Br J Nutr. (2016) 115:842–50. doi: 10.1017/S000711451500519X 26787123

[22] Sadeghi O Keshteli AH Afshar H Esmaillzadeh A Adibi P. Adherence to Mediterranean dietary pattern is inversely associated with depression anxiety and psychological distress. Nutr Neurosci. (2021) 24:248–59. doi: 10.1080/1028415X.2019.1620425, PMID: 31185883

[23] Hutchins-Wiese HL Bales CW Starr KNP. Mediterranean diet scoring systems: understanding the evolution and applications for Mediterranean and non-Mediterranean countries. Br J Nutr. (2021): 1-22) 128:1371–92. doi: 10.1017/S0007114521002476 34289917

[24] Khan R Waqas A Bilal A Mustehsan ZH Omar J Rahman A. Association of Maternal Depression with diet: a systematic review. Asian J Psychiatr. (2020) 52:102098. doi: 10.1016/j.ajp.2020.102098, PMID: 32403029

[25] Kim Y Je Y. A modified Mediterranean diet score is inversely associated with metabolic syndrome in Korean adults. Eur J Clin Nutr. (2018) 72:1682–9. doi: 10.1038/s41430-018-0156-4, PMID: 29563642

[26] Fung TT McCullough ML Newby P Manson JE Meigs JB Rifai N. Diet-quality scores and plasma concentrations of markers of inflammation and endothelial dysfunction. Am J Clin Nutr. (2005) 82:163–73. doi: 10.1093/ajcn/82.1.163, PMID: 16002815

[27] Kim DW Song S Lee JE Oh K Shim J Kweon S. Reproducibility and validity of an Ffq developed for the Korea National Health and nutrition examination survey (Knhanes). Public Health Nutr. (2015) 18:1369–77. doi: 10.1017/S1368980014001712, PMID: 25167205 PMC10271806

[28] Lee JE McLerran DF Rolland B Chen Y Grant EJ Vedanthan R. Meat intake and cause-specific mortality: a pooled analysis of Asian prospective cohort studies. Am J Clin Nutr. (2013) 98:1032–41. doi: 10.3945/ajcn.113.062638, PMID: 23902788 PMC3778858

[29] Rural Development Administration, National Academy of Agricultural Science. 8th revision standard food composition table. (2013). Available at: http://koreanfood.rda.go.kr/eng/fctFoodSrchEng/main.

[30] Kroenke K Spitzer RL Williams JB. The Phq-9: validity of a brief depression severity measure. J Gen Intern Med. (2001) 16:606–13. doi: 10.1046/j.1525-1497.2001.016009606.x, PMID: 11556941 PMC1495268

[31] Park S-J Choi H-R Choi J-H Kim K-W Hong J-P. Reliability and validity of the Korean version of the patient health Questionnaire-9 (Phq-9). Anxiety Mood. (2010) 6:119–24.

[32] Moussavi S Chatterji S Verdes E Tandon A Patel V Ustun B. Depression, chronic diseases, and decrements in health: results from the world health surveys. Lancet. (2007) 370:851–8. doi: 10.1016/S0140-6736(07)61415-9, PMID: 17826170

[33] Oddo VM Welke L McLeod A Pezley L Xia Y Maki P. Adherence to a Mediterranean diet is associated with lower depressive symptoms among us adults. Nutrients. (2022) 14:278. doi: 10.3390/nu14020278, PMID: 35057462 PMC8780598

[34] Jacka FN O’Neil A Opie R Itsiopoulos C Cotton S Mohebbi M. A randomised controlled trial of dietary improvement for adults with major depression (the ‘Smiles’trial). BMC Med. (2017) 15:1–13. doi: 10.1186/s12916-017-0791-y 28137247 PMC5282719

[35] Bayes J Schloss J Sibbritt D. The effect of a Mediterranean diet on the symptoms of depression in young males (the “Ammend: a Mediterranean diet in men with depression” study): a randomized controlled trial. Am J Clin Nutr. (2022) 116:572–80. doi: 10.1093/ajcn/nqac106, PMID: 35441666

[36] Fernstrom JD. Effects of dietary polyunsaturated fatty acids on neuronal function. Lipids. (1999) 34:161–9. doi: 10.1007/s11745-999-0350-3 10102242

[37] Jeon BY Seo HN Yun A Lee IH Park DH. Effect of glasswort (*Salicornia Herbacea* L.) on Nuruk-making process and Makgeolli quality. Food Sci Biotechnol. (2010) 19:999–1004. doi: 10.1007/s10068-010-0140-9

[38] Pandey KB Rizvi SI. Plant polyphenols as dietary antioxidants in human health and disease. Oxidative Med Cell Longev. (2009) 2:270–8. doi: 10.4161/oxim.2.5.9498, PMID: 20716914 PMC2835915

[39] Behl T Rana T Alotaibi GH Shamsuzzaman M Naqvi M Sehgal A. Polyphenols inhibiting Mapk Signalling pathway mediated oxidative stress and inflammation in depression. Biomed Pharmacother. (2022) 146:112545. doi: 10.1016/j.biopha.2021.112545, PMID: 34922112

[40] Gomez-Pinilla F Nguyen TT. Natural mood foods: the actions of polyphenols against psychiatric and cognitive disorders. Nutr Neurosci. (2012) 15:127–33. doi: 10.1179/1476830511Y.0000000035, PMID: 22334236 PMC3355196

[41] Yu H Chen Z-y. The role of Bdnf in depression on the basis of its location in the neural circuitry. Acta Pharmacol Sin. (2011) 32:3–11. doi: 10.1038/aps.2010.184, PMID: 21131999 PMC4003317

[42] Altemus M Sarvaiya N Epperson CN. Sex differences in anxiety and depression clinical perspectives. Front Neuroendocrinol. (2014) 35:320–30. doi: 10.1016/j.yfrne.2014.05.004, PMID: 24887405 PMC4890708

[43] Faas MM Melgert BN de Vos P. A brief review on how pregnancy and sex hormones interfere with taste and food intake. Chemosens Percept. (2010) 3:51–6. doi: 10.1007/s12078-009-9061-5, PMID: 20352054 PMC2844535

[44] Freeman EW Sammel MD Lin H Nelson DB. Associations of hormones and menopausal status with depressed mood in women with no history of depression. Arch Gen Psychiatry. (2006) 63:375–82. doi: 10.1001/archpsyc.63.4.375 16585466

[45] Klein SL. The effects of hormones on sex differences in infection: from genes to behavior. Neurosci Biobehav Rev. (2000) 24:627–38. doi: 10.1016/S0149-7634(00)00027-0, PMID: 10940438

[46] Frokjaer VG Pinborg A Holst KK Overgaard A Henningsson S Heede M. Role of serotonin transporter changes in depressive responses to sex-steroid hormone manipulation: a positron emission tomography study. Biol Psychiatry. (2015) 78:534–43. doi: 10.1016/j.biopsych.2015.04.015, PMID: 26004162

[47] Gaskin JL Pulver AJ Branch K Kabore A James T Zhang J. Perception or reality of body weight: which matters to the depressive symptoms. J Affect Disord. (2013) 150:350–5. doi: 10.1016/j.jad.2013.04.017 23706878

[48] Hong JW Noh JH Kim D-J. The prevalence of and factors associated with depressive symptoms in the Korean adults: the 2014 and 2016 Korea National Health and nutrition examination survey. Soc Psychiatry Psychiatr Epidemiol. (2021) 56:659–70. doi: 10.1007/s00127-020-01945-2, PMID: 32780175

[49] Akhtar-Danesh N Landeen J. Relation between depression and sociodemographic factors. Int J Ment Heal Syst. (2007) 1:4–9. doi: 10.1186/1752-4458-1-4 PMC2241832 18271976

[50] Chunnan L Shaomei S Wannian L. The association between sleep and depressive symptoms in us adults: data from the Nhanes (2007–2014). Epidemiol Psychiatr Sci. (2022) 31:e63. doi: 10.1017/S2045796022000452, PMID: 36073029 PMC9483824

[51] Carter JD Joyce PR Mulder RT Luty SE McKenzie J. Gender differences in the presentation of depressed outpatients: a comparison of descriptive variables. J Affect Disord. (2000) 61:59–67. doi: 10.1016/S0165-0327(00)00151-8, PMID: 11099741

[52] Jung IH Jung MA Kim EJ Han M Kim DH. *Lactobacillus Pentosus* Var. Plantarum C29 protects scopolamine-induced memory deficit in mice. J Appl Microbiol. (2012) 113:1498–506. doi: 10.1111/j.1365-2672.2012.05437.x, PMID: 22925033

[53] Yue X Li M Liu Y Zhang X Zheng Y. Microbial diversity and function of soybean paste in East Asia: what we know and what we Don’t. Curr Opin Food Sci. (2021) 37:145–52. doi: 10.1016/j.cofs.2020.10.012

[54] Selhub EM Logan AC Bested AC. Fermented foods, microbiota, and mental health: ancient practice meets nutritional psychiatry. J Physiol Anthropol. (2014) 33:1–12. doi: 10.1186/1880-6805-33-2 24422720 PMC3904694

[55] Pulikkan J Mazumder A Grace T. Role of the gut microbiome in autism Spectrum disorders. Rev. Biomark. Stud. Psychiatr. Neurodegenerat. Disord. (2019) 1118:253–69. doi: 10.1007/978-3-030-05542-4_13 30747427

[56] Coates J Colaiezzi B Fiedler JL Wirth J Lividini K Rogers B. A program needs-driven approach to selecting dietary assessment methods for decision-making in food fortification programs. Food Nutr Bull. (2012) 33:S146–56. doi: 10.1177/15648265120333S202 23193765

[57] Christiansen M Vik PW Jarchow A. College student heavy drinking in social contexts versus alone. Addict Behav. (2002) 27:393–404. doi: 10.1016/S0306-4603(01)00180-0, PMID: 12118627

[58] Ju YJ Kim W Oh SS Park E-C. Solitary drinking and the risk of depressive symptoms and suicidal ideation in college students: findings from a Nationwide survey in Korea. J Affect Disord. (2019) 257:710–5. doi: 10.1016/j.jad.2019.07.080, PMID: 31382123

